# Early childhood low parental income and the risk of mental disorder in adolescence and early adulthood. A register study of migrants and non-migrants

**DOI:** 10.1192/j.eurpsy.2022.1621

**Published:** 2022-09-01

**Authors:** K. Hynek, M. Straiton

**Affiliations:** Norwegian Institute of Public Health, Department Of Mental Health And Suicide, Oslo, Norway

**Keywords:** Low parental income, migrants, mental disorder, Outpatient mental healthcare

## Abstract

**Introduction:**

Low parental income during childhood is associated with increased risk of mental disorders at later ages. However, despite a disproportionate share of migrant children growing up in persistent poverty, as compared to their majority counterparts, the research on whether the association varies by migrant background is limited.

**Objectives:**

Is there an association between parental income during early childhood and the risk of mental disorder, defined by use of outpatient mental healthcare services (OPMH), in adolescence and early adulthood? Does the strength of the association vary by migrant background?

**Methods:**

Information from five national registers were combined to study a population of 577,072 individuals. We applied discrete-time logistic regression, with an interaction term between parental income and migrant background to study differences in the association by migrant background.

**Results:**

Low parental income during early childhood was associated with twice the odds of OPMH use in adolescence and early adulthood compared to individuals with higher parental income. Even after adjusting for a range of covariates the association remained significant, yet, weaker. The association was, however, in the opposite direction for migrants. Those in the higher income group had higher probability of OPMH use. The relative differences within groups were small, but significant for migrants from Middle East and North Africa, South Asia and Western countries.

**Conclusions:**

Social inequalities in mental health may have an onset already in childhood, Therefore, future interventions should focus on reducing social inequalities in childhood in order to improve the mental health in young people.

**Disclosure:**

No significant relationships.

